# 

**Published:** 1898-11

**Authors:** 


### CONTENTS.

ORIGINAL COMMUNICATIONS. PAGE

Some Reflex Disorders of Dental Origin. By HENRY H. BURCHARD, M.D., D.D.S., Philadelphia . . . . . 697
Removal of the Dental Pulp. By DR. B. HOLLY SMITH . . . . . 702
What Instruments shall I carry? By J. T. CODMAN, D.M.D., Boston, Mass. . . 704
Reminiscences of a European Trip to the International Medical Congress at Moscow, Russia, August 19 to 26, 1897, as a Delegate from the American Medical Association and the Odontological Society of Pennsylvania. (*Continued.*) By W. G. A. BONWILL, D.D.S., Philadelphia . . . . . 707

ABSTRACTS AND TRANSLATIONS.

Abstract of the Report of the Committee on Foreign Relations of the National Association of Dental Faculties . . . . . 713
Gold Linings and Strengtheners for Vulcanite Plates. By J. H. GARTRELL . . . 724

REPORTS OF SOCIETY MEETINGS.

National Dental Association . . . . . 728

American Academy of Dental Science . . . . . 732

Academy of Stomatology . . . . . 745

EDITORIAL.

Report of Committee on Foreign Degrees . . . . . 750

BIBLIOGRAPHY . . . . . 753

OBITUARY.

Dr. Gardner Quincy Colton . . . . . 754
Dr. Emile de Trey . . . . . 755

NOTES AND COMMENTS . . . . . 756

CURRENT NEWS.

Southern California Dental Association . . . . . 759

